# The Role of CDR1as in Proliferation and Differentiation of Human Umbilical Cord-Derived Mesenchymal Stem Cells

**DOI:** 10.1155/2019/2316834

**Published:** 2019-06-12

**Authors:** Lunyu Yang, Zhang Bin, Shi Hui, Li Rong, Benshuai You, Peipei Wu, Xinye Han, Hui Qian, Wenrong Xu

**Affiliations:** ^1^Zhenjiang Key Laboratory of High Technology Research on Exosomes Foundation and Transformation Application, Jiangsu Key Laboratory of Medical Science and Laboratory Medicine, School of Medicine, Jiangsu University, Zhenjiang, Jiangsu, China; ^2^Institute of Forensic Medicine and Laboratory Medicine, Jining Medical University, Jining, Shandong, China

## Abstract

Mesenchymal stem cells derived from human umbilical cord (hucMSCs) are considered a promising tool for regenerative medicine. circRNAs as newly discovered noncoding RNAs are involved in multiple biological processes. However, little has been known about the function of circRNAs in the proliferation and differentiation of hucMSCs. In this study, we selected several circRNAs expressed in MSCs from circBase and found that CDR1as expression level was markedly significant. We observed that, compared with that of uninduced hucMSCs, the CDR1as expression level of induced hucMSCs decreased with cell induction differentiation. By using siRNA to knock down CDR1as of hucMSCs, we discovered that proliferation was inhibited but the apoptosis increased. In addition, we found that the expression of stemness transcription factors (STFs) was downregulated after CDR1as knockdown and the adipogenesis and osteogenesis potential of hucMSCs was impaired. Our findings suggest that CDR1as takes a part in maintaining proliferation and differentiation of hucMSCs, providing clues for MSC modification and further for stem cell therapy and tissue regeneration.

## 1. Introduction

Mesenchymal stem cells (MSCs) are a group of multipotent stem cells with characteristics of self-renewal and multipotency to differentiate into adipocytes, osteoblasts, chondrocytes, and other lineage cells [1]. To date, MSCs have been isolated from various tissues including bone morrow, endodontic pulp, umbilical cord, hair follicle, adipose, and other tissues [2–6]. Due to their proliferation and differentiation potential as well as the established protocols for their isolation and propagation *in vitro*, MSCs have become attractive tools for tissue injury repair and regenerative medicine [7]. For example, engrafted MSCs could differentiate into tissue-specific cells or function-related cells required for tissue regeneration [8, 9]. On the other hand, MSCs could secrete a variety of bioactive molecules for anti-inflammatory, antiapoptotic, angiogenesis, and immunomodulatory effects in injury repair [10–13]. Although *in vitro* and *in vivo* studies have indicated that MSCs played a powerful role in tissue injury repair, the molecular mechanism that regulates the proliferation and differentiation of MSCs has not yet been clarified.

Circular RNAs (circRNAs), a novel member of noncoding RNAs, are abundant and extensively expressed in mammals [14, 15]. High-throughput sequencing technology has revealed that circRNAs are expressed in a cell type- and cell stage-specific manner, serving a critical role in eukaryotes [15, 16]. So far, circRNAs have been found to function in three major ways, that is, as microRNA sponges to sequester and inhibit miRNA activity, as regulators to interact with proteins, and as working templates to synthesize proteins [16–19]. Through those functions, circRNAs take an important part in many biological processes. For instance, circHIPK3, an abundant circRNA derived from the HIPK3 gene, regulates cell growth by sponging several miRNAs [20]. Circ-Foxo3, mainly located in the cytoplasm, could interact with ID-1, E2F1, FAK, and HIF1alpha proteins to promote cardiac senescence [21]. CircRNA-2837 was reported to regulate neuronal autophagy by sponging the miR-34 family [22]. Moreover, circBIRC6, which is highly expressed in undifferentiated human embryonic stem cells (hESCs), enables the maintenance of hESC pluripotency by interacting with miR-34a and miR-145 [23]. Despite the accumulated knowledge above, the underlying mechanism with regard to the exact role of circRNAs in MSC biology remains unclear. Herein, we selected several candidate circRNAs, including CDR1as, circBIRC6, circSMARCA5, circASXL1, and circHIPK3 from circBase, and investigated their functional roles in human umbilical cord mesenchymal stem cells (hucMSCs). By identifying CDR1as that was highly expressed in hucMSCs, we found that CDR1as was involved in regulating the proliferation and differentiation of hucMSCs, showing its great potential in clinical applications.

## 2. Materials and Methods

All experimental protocols were approved by the Medical Ethics of Jiangsu University (2012258).

### 2.1. Cell Culture

hucMSCs were isolated and identified as previously described [4]. hucMSCs were cultured in *α*-MEM medium (Invitrogen) with 10% fetal bovine serum (Bioind) and were used for experiments in 3 to 5 passages. 293 T cells were purchased from ATCC and cultured in high DMEM (Gibco) containing 10% fetal bovine serum (Bovogen). All cells were maintained at 37°C with 5% CO_2_.

### 2.2. DIM-Treated hucMSCs

DIM was prepared to treat hucMSCs as described before [24]. DIM was dissolved in dimethyl sulfoxide (DMSO) to prepare a 500 mM stock solution and diluted to 50 *μ*M in *α*-MEM as a working solution. The control group was treated with *α*-MEM medium containing 0.1% DMSO. After 48h, total RNA was collected to detect the changes of gene expression.

### 2.3. RNA Interference

Small interfering RNA (siRNA) targeting the back-splice junction of CDR1as was synthesized by GenePharma (Shanghai, China). The sequence of siRNA was as follows: sense, 5′-UAUCCAGGGUUUCCGAUGGTT-3′; antisense, 5′-CCAUCGGAAACCCUCCAUATT-3′. SiRNA with final concentration of 50 nM was transfected into hucMSCs and 239 T cells using the Lipofectamine 2000 reagent (Life Technologies) according to the manufacturer's instructions.

### 2.4. Western Blot

Cells were lysed in RIPA (Pierce) buffer containing proteinase inhibitors (Pierce). An equal amount of proteins was separated on 12% SDS-polyacrylamide gel. After electrophoresis, proteins were transferred to a PVDF membrane, then blocked with 5% nonfat milk and incubated with primary antibodies against *β*-actin (Bioworld), PCNA (SAB), Bcl-2 (CST), Bax (CST), caspase 9 (CST), activated caspase 3 (Santa Cruz), Oct4 (CST), Sox2 (CST), Lin28 (SAB), Nanog (SAB), and Klf4 (CST) at 4°C overnight. Membrane was washed for three times with TBST and subsequently incubated with HRP-conjugated goat anti-rabbit or anti-mouse secondary antibodies (Bioworld) at 37°C for 1 hour. The bands were visualized by using an enhanced chemiluminescence.

### 2.5. Real-Time Quantitative RT-PCR

The total RNA from cells was extracted using TRIzol reagent (Glibo) and the concentration was measured by NanoDrop One (Thermo Fisher Scientific). 1 *μ*g of RNA was reverse transcribed to cDNA by using reverse transcriptase (Vazyme). Oligo dT and random primers were used to synthesize cDNA of linear and circular RNAs, respectively. Real-time quantitative PCR was performed by using the AceQ qPCR SYBR Green Master Mix (Vazyme) on a Bio-Rad CFX96 Detection System; *β*-actin was used as qRT-PCR control. The primers for target genes are listed in Table 1. All experiments were performed in triplicate and the relative quantification of gene expression was performed by using 2^-ΔΔCT^ method [25].

### 2.6. Colony Formation Assay

Twenty-four hours after transfection, hucMSCs were seeded into 6-well plates with 1500 cells per well and maintained for 10 days. Mediums were replaced every three days during the whole experiment. Then, the cells were fixed with 4% paraformaldehyde for 30 min and stained with 1% crystal violet for 5 min at room temperature. The number of colonies was photographed and counted.

### 2.7. CCK-8 Assay

CCK-8 assay was performed every 24 h after knocking down CDR1as. hucMSCs were seeded into 96-well plates with 1500 cells per well and treated with 10 *μ*l CCK-8 reagent (Vazyme, Nanjing, China) according to the manufacturer's protocol. After being incubated 2-4 hours, the citation imaging reader (BioTek, USA) was used to analyze the absorbance values at 450 nm.

### 2.8. Cell Cycle Analysis

Seventy-two hours after transfection, cells were fixed in ice-cold 70% ethanol for 24 h and stained with propidium iodide (PI) master mix (FCMRCS) at 37°C for 30 min according to the manufacturer's protocol before their analysis using flow cytometry. The results were analyzed by the ModFit LT software.

### 2.9. Cell Apoptosis Analysis

Seventy-two hours after transfection, cell apoptosis was analyzed using the Annexin V-FITC/Propidium Iodide (PI) Apoptosis Detection Kit (Invitrogen) according to the manufacturer's instruction. hucMSCs were stained with Annexin V and PI and detected by using flow cytometry.

### 2.10. Osteogenic and Adipogenic Differentiation

hucMSCs were seeded in 6-well plates, transfected or not, with adipogenic differentiation medium (Cyagen Biosciences, CA, USA) or osteogenic differentiation medium (Cyagen Biosciences, CA, USA) for about 2 weeks according to the manufacturer's protocols. After the induction, the potential of adipogenic and osteogenic differentiation was evaluated through oil red O and alizarin red staining, respectively.

### 2.11. Statistical Analysis

All data were analyzed by GraphPad Prism software (version 5.0) and shown as mean ± SD. One-way ANOVA or *t*-tests were performed to analyze the differences between groups. *P* ≤ 0.05 was considered statistically significant. Asterisks (^∗^, ^∗∗^, and ^∗∗∗^) stand for *P* < 0.05, *P* < 0.001, and *P* < 0.0001, respectively.

## 3. Results

### 3.1. CDR1as Was Highly Expressed in hucMSCs

**Figure 1:**
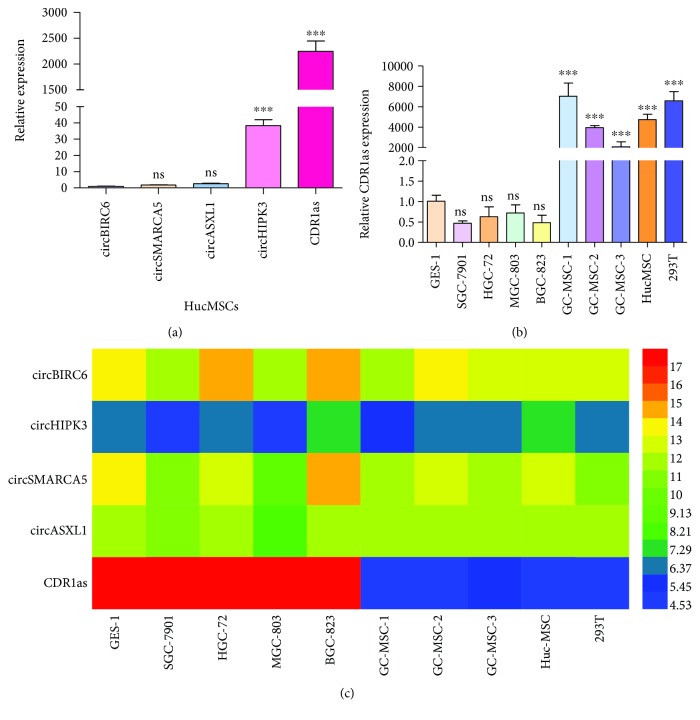
CDR1as was highly expressed in hucMSCs. (a) Using RT-qPCR, the relative expression levels of circRNAs in hucMSCs were detected. (b) RT-qPCR was used to analyze the expression level of CDR1as in different cells. (c) Heat map of RT-qPCR data (relative expression values ΔCT) for showing the expression levels of circRNAs in different cell lines. CircRNA with a higher expression level is mapped to the blue part and a lower expression level is mapped to the red part. ns: no significance; ^∗^ *P* < 0.05; ^∗∗^ *P* < 0.01; ^∗∗∗^ *P* < 0.001.

To explore the role of circRNAs in MSCs, we first selected several circRNAs which were expressed in MSCs from circBase [26] (Supplementary Table 1). We then detected the expression levels of five circRNAs including CDR1as, circBIRC6 [23], circSMARCA5 [19, 27, 28], circASXL1, and circHIPK3 [20], most of which have been reported to regulate cell proliferation and tumor growth. We found that, compared with other circRNAs, CDR1as was much more abundant in hucMSCs (Figure 1(a)). To further investigate the expression level of CDR1as in other cells, we collected RNAs from gastric cancer cells, gastric epithelial cells, MSCs isolated from gastric cancer tissue and human umbilical cord, and 293 T cells. Compared with cancer cells or epithelial cell, CDR1as was highly expressed in MSCs with significance, and we found that CDR1as expression level in 293 T cells was similar to that in MSCs (Figure 1(b)). In addition, we also detected the expression of other four circRNAs in all cells, the results of which showed that the circHIPK3 expression level on the whole was higher than the expression level of circBIRC6, circSMARCA5, or circASXL1. However, its expression level was not as abundant as that of CDR1as in MSCs (Figure 1(c)). Furthermore, the expression of circHIPK3, circBIRC6, circSMARCA5, or circASXL1 was not as significant in the difference between cells as that of CDR1as (Supplementary Figure 1). Collectively, these primitive results suggested that CDR1as may play an important role in MSC biology.

### 3.2. CDR1as May Be a Key Factor in Regulation of hucMSC Differentiation

**Figure 2:**
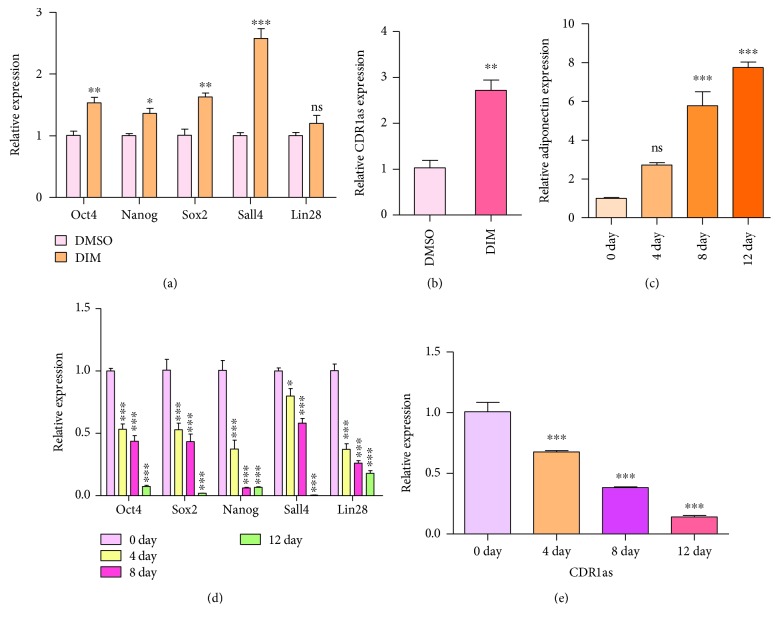
CDR1as expression level varied with the state of hucMSCs. (a) The expression levels of STFs were detected by RT-qPCR after the DIM treatment of hucMSCs. (b) After treating hucMSCs with DIM, CDR1as expression was detected by RT-qPCR. (c) The expression level of adiponectin was detected by RT-qPCR in the adipogenic differentiation of hucMSCs. (d) The expression levels of STFs were detected by RT-qPCR with hucMSC adipogenic differentiation. (e) The expression level of CDR1as was detected by RT-qPCR in the adipogenic differentiation of hucMSCs. ns: no significance; ^∗^ *P* < 0.05; ^∗∗^ *P* < 0.01; ^∗∗∗^ *P* < 0.001.

Our previous work had demonstrated that small molecular drug DIM treatment upregulated the expression of stemness transcription factors (STFs) and enhanced the differentiation ability of hucMSCs [24]. We therefore examined whether the expression level of CDR1as could be changed after DIM treatment. As a result, we noticed that the expression levels of CDR1as and STFs were both upregulated in hucMSCs after DIM treatment (Figures 2(a) and 2(b)), suggesting that as a key factor, CDR1as may regulate the proliferation and differentiation of hucMSCs. To further study whether CDR1as expression varied during cell differentiation, the expression of adiponectin as the marker of adipocytes was upregulated in hucMSCs after adipogenic differentiation induction (Figure 2(c)). The results showed that when cells differentiated, multiple STFs (i.e., Oct4, Nanog, Sox2, Sall4, and Lin28) were downregulated over time (Figure 2(d)). Simultaneously, the expression of CDR1as also decreased gradually as cells underwent adipogenic differentiation (Figure 2(e)). These results revealed that with relevancy to STFs, CDR1as may function significantly in the differentiation of hucMSCs.

### 3.3. Knockdown of CDR1as Inhibited Cell Proliferation and Induced Cell Apoptosis

**Figure 3:**
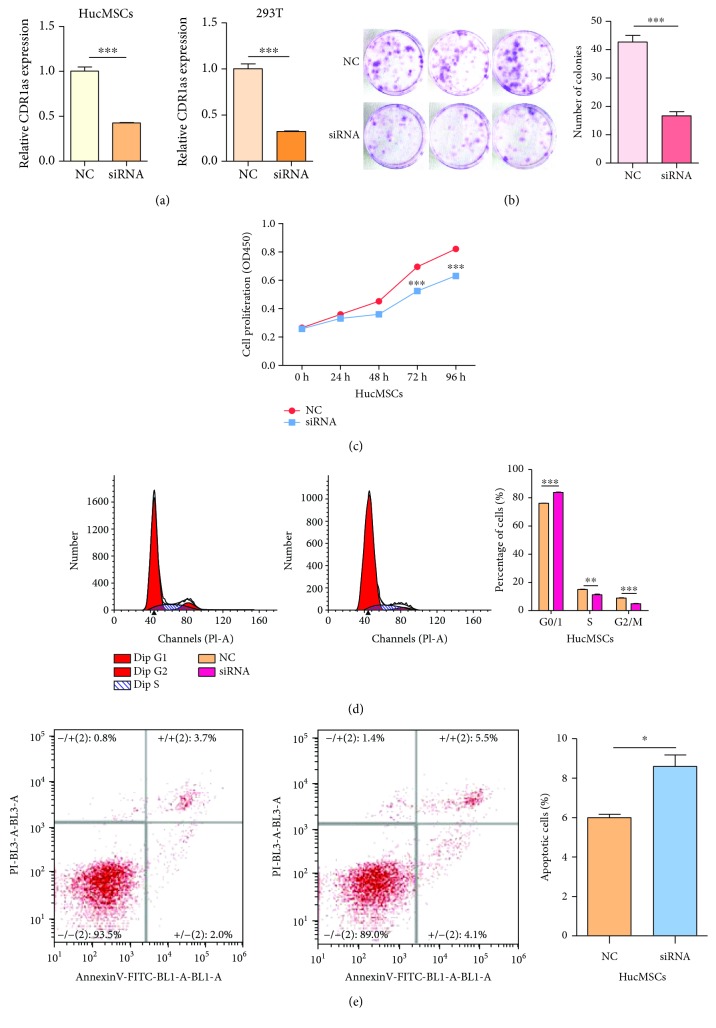
Knockdown of CDR1as inhibited cell proliferation and induced cell apoptosis. (a) The knockdown efficiency of CDR1as in hucMSCs and 293 T cells. (b) Colony-forming assay of hucMSCs transfected with siRNA of CDR1as or negative control. (c, d) CCK-8 and cell cycle assay of hucMSCs transfected with siRNA of CDR1as or negative control to evaluate cell proliferative ability. (e) Flow cytometry apoptosis analysis of hucMSCs transfected with siRNA or control. ^∗^ *P* < 0.05; ^∗∗^ *P* < 0.01; ^∗∗∗^ *P* < 0.001.

Next, we employed RNA interference to alter the expression of CDR1as. We designed one small interfering RNA (siRNA) that targeted the back-spliced sequence of CDR1as. As predicted, this siRNA knocked down CDR1as effectively in hucMSCs and 293 T cells (Figure 3(a)). Subsequently, results from cell colony formation assay indicated that the downregulation of CDR1as significantly suppressed the cell growth of hucMSCs (Figure 3(b)). A CCK-8 assay also confirmed that the proliferation of hucMSCs was impaired by CDR1as knockdown. (Figure 3(c)). Moreover, cell cycle assay showed that CDR1as knockdown led to cell arrest at G0/G1 phase (Figure 3(d)). Then, we examined the effect of CDR1as knockdown on cell apoptosis. After the knockdown of CDR1as in hucMSCs, we found that the proportion of apoptotic cells became higher than that in the control group (Figure 3(e)).

**Figure 4:**
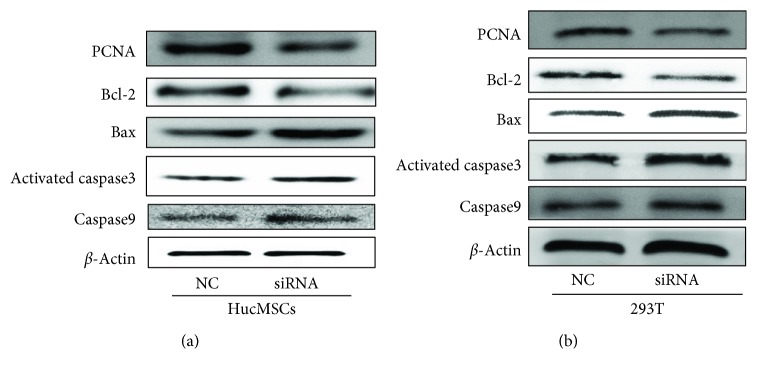
The expression changes of proliferative and apoptotic-related proteins. (a) Western blot for PCNA, Bcl-2, Bax, activated-caspase 3, and caspase 9 expression in hucMSCs after transfection with siRNA or control. (b) Western blot for PCNA, Bcl-2, Bax, activated-caspase 3, and caspase 9 expression in 293 T after transfection with siRNA or control.

At the same time, we found that the protein level of Proliferating Cell Nuclear Antigen (PCNA) in hucMSCs and 293 T cells decreased after the knockdown of CDR1as (Figures 4(a) and 4(b)). Both in hucMSCs and 293 T cells after CDR1as knockdown, B-cell lymphoma-2 (Bcl-2) decreased, while the expression level of Bax increased. Moreover, activated caspase 3 and caspase 9 increased, too (Figures 4(a) and 4(b)). Put together, these findings suggested that CDR1as knockdown inhibited cell proliferation and induced cell apoptosis.

### 3.4. Knockdown of CDR1as Inhibited the Differentiation of hucMSCs

**Figure 5:**
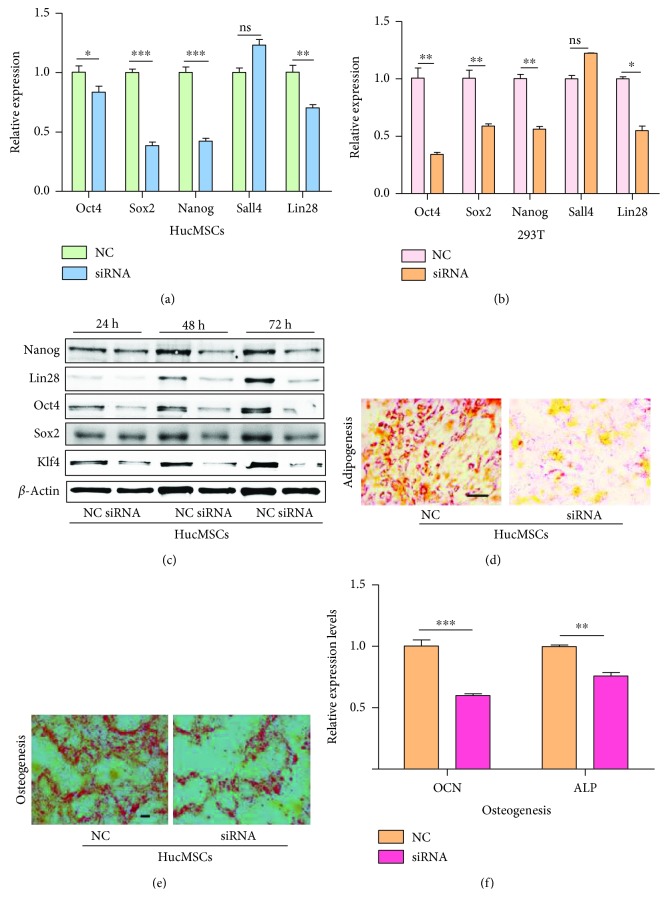
Knockdown of CDR1as inhibited the differentiation of hucMSCs. (a) The expression levels of STFs in hucMSCs transfected with siRNA or control. (b) The expression levels of STFs in 293 T cells transfected with siRNA or control. (c) Western blot analysis for the expression of Oct4, Lin28, Nanog, Klf4, and Sox2 in hucMSCs transfected with siRNA or control for 24 h, 48 h, and 72 h, respectively. (d) Adipogenic differentiation of hucMSCs after transfection with siRNA or control. (e) Osteogenic differentiation of hucMSCs after transfection with siRNA or control. (f) RT-qPCR for detecting the expression levels of OCN and ALP. ns: no significance; ^∗^ *P* < 0.05; ^∗∗^ *P* < 0.01; ^∗∗∗^ *P* < 0.001.

To analyze the effect of CDR1as on the differentiation of hucMSCs, we first examined the expression level of STFs, with a finding that the mRNA levels of Oct4, Sox2, Nanog, and Lin28 were downregulated in both hucMSCs and 293 T cells after the knockdown of CDR1as, while the level of Sall4 did not change significantly (Figures 5(a) and 5(b)). We also found that the protein levels of Oct4, Lin28, Nanog, Sox2, and Klf4 decreased after the knockdown of CDR1as in hucMSCs (Figure 5(c)). Then, we examined how the differentiation potential of hucMSCs could be affected after knocking down CDR1as. As a result, it showed that the ability of hucMSCs to differentiate into adipocytes or osteocytes was impaired after CDR1as knockdown (Figures 5(d) and 5(e)). And the expression of OCN and ALP, as markers of osteocytes, was found to decrease in the siRNA treatment group (Figure 5(f)). Taken together, these findings pointed out that CDR1as helped maintain the pluripotency of hucMSCs.

## 4. Discussion

Owing to high-throughput sequencing technology development, thousands of circRNAs were identified in eukaryotes, with many more to come continuously [14, 16]. However, little is known about the role of circRNAs in MSCs. In our study, we selected several candidates circRNAs including CDR1as, circBIRC6 [23], circSMARCA5 [19, 27, 28], circASXL1, and circHIPK3 [20] from circBase [26], which might regulate the biological functions of MSCs. As a result, we found that CDR1as stands out. Currently, the exact function of CDR1as in MSCs still remains unknown. CDR1as, one of the extensively studied circular RNAs, was initially identified to be abundant in brain tissues and harbors more than 60 conserved sites for miR-7 [16, 29]. Although CDR1as was found to function via sponging multiple microRNAs, until now, only miR-671 can directly cleave CDR1as in an Ago2-slicer-dependent manner [30–32]. In addition to affecting the development and function of the brain, CDR1as was also reported to promote the progression of multiple tumors via sponging miR-7 [33–37]. On the other hand, CDR1as exerted an anticancer effect in bladder cancer via sponging miR-135a [38]. Moreover, overexpressed CDR1as in islet cells increased the secretion of insulin via CDR1as/miR-7 axis [39]. Apart from the CDR1as reported widely, several other circRNAs also have been reported in previous researches. For example, circBIRC6 was reported to maintain the pluripotency of human embryonic stem cells (hESCs) via sponging miR-34a and miR-145 [23]. CircSMARCA5 was reported to inhibit the migration of glioblastoma multiforme cells but promotes the proliferation of prostate cancer cells [19, 27, 28]. CircHIPK3 was also reported to promote the progression of multiple cancers by sponging several miRNAs [40–43]. However, none of those circRNAs has been studied in MSCs.

In this study, we first found that the expression level of CDR1as was abundant in hucMSCs. More experiments revealed that CDR1as was highly expressed not only in hucMSCs but also in MSCs from gastric cancer tissues of three patients as well as 293 T cell. Our previous studies have shown that small molecular drug 3,3′-diindolylmethane (DIM) could improve the stemness of hucMSCs and enhance their proliferation, differentiation, and paracrine abilities by activating Wnt/*β*-catenin signal axis [24]. Consistent with previous results, we found that after the treatment of hucMSCs with DIM, the expression levels of STFs were upregulated. Simultaneously, we also found that CDR1as expression was also upregulated after the treatment of hucMSCs with DIM. In addition, we found that CDR1as as well as STFs was downregulated within hucMSC adipogenesis. STFs, such as Oct4, Sox2, and Nanog, are indispensable for the maintenance of pluripotency in hESCs [44]. In addition to STFs, noncoding RNAs including circRNAs have also been shown to have important roles in pluripotency maintenance. For example, by interacting with miR-34a and miR-145, which are known to repress the expression of pluripotency-associated genes, circBIRC6 participates in maintaining the pluripotency of hESCs [23]. Meanwhile, the biogenesis of circBIRC6 is promoted by the splicing factor ESRP1, whose expression is controlled by the transcription factors Oct4 and Nanog [23]. Therefore, a molecular circuitry is formed between the circRNA and the STFs. In our study, we found that the CDR1as and STF expression changed in the same direction. However, the regulatory mechanism of CDR1as expression changes still needs more in-depth study.

Loss-of-function experiments revealed that CDR1as knockdown inhibited hucMSC proliferation, downregulated the expression of STFs, and impaired their differentiation potential. Cell proliferation and multiple differentiation potential are important characteristics of MSCs, which also are the important basis for MSCs to be used in damage repair. In long regular culture, MSCs will undergo spontaneous osteogenic differentiation and STFs such as Oct4 and Sox2 expressions will decline over time, which are associated with the epigenetic dysregulation of histone H3 acetylation in K9 and K14 [45]. So far, a series of studies have shown that noncoding RNAs, especially microRNAs, have important roles in regulating the proliferation and differentiation of stem cells. For example, miR-21 inhibits the proliferation of MSCs by directly inhibiting the expression of Sox2 [46]. MiR-145 and miR-34a are known to repress STF expression to promote the differentiation of hESCs [47, 48]. Moreover, miR-7, which enjoys more than 60 binding sites on CDR1as [16, 29], has been demonstrated to inhibit the proliferation of cancer stem-like cells isolated from breast cancer via modulating Klf4 [49]. Current studies have shown that most circRNAs play their biological roles mainly by acting as the sponges of microRNAs [50]. However, whether CDR1as functions in hucMSCs through sponging miR-7 or other miRNAs needs to be investigated.

Although MSCs provide a promising cell source for tissue regeneration, two shortcomings hinder its clinical applications. First, MSCs lose proliferation and differentiation potential upon expansion *in vitro* [51, 52], so a continuous isolation of MSCs from tissues is required. Second, the number and quality of cells from tissues decreased as the donor age increased [53, 54]. Currently, the ways to improve the therapeutic efficacy of MSCs mainly include three aspects: (i) search for new biomaterials to culture MSCs *in vitro*; (ii) modification of MSCs with small-molecule compounds; and (iii) genetic modification of MSCs. When the cells were isolated from tissues, studies have demonstrated that, compared with the conventional culture systems, cells cultured on biomaterials like type I collagen [55] and chitosan film [56, 57] could maintain the stemness and differentiation potential of MSCs. Moreover, small molecular compounds also are popular tools used to modulate stem cell function for its convenience [58]. Our previous work showed that small-molecule compounds 3,3′-diindolylmethane not only promoted the proliferation but also upregulated the stemness of hucMSCs *in vitro*, thereby improving the effect of hucMSCs for wound healing *in vivo* [24]. In addition, researchers find that genetic manipulation also is an effective way to modify MSCs. Pluripotency-associated transcription factors like Oct4, Sox2, and Nanog expressed in MSCs at low levels were introduced into MSCs, therefore enhancing their proliferative activity and multilineage differentiation potential [59, 60]. Moreover, the overexpression of IGF-1, which was involved in regulating proliferation and differentiation, increased the cell survival and osteogenic potential of aging MSCs [61]. And overexpressed IGF-1 was demonstrated to promote fracture healing and new bone formation *in vivo* [62]. With more findings revealing that noncoding RNAs are regulators in MSC biological processes and therapeutic actions [63, 64], our results here may provide a hint for the potential application of circRNAs in regenerative medicine.

## 5. Conclusions

Our findings revealed that CDR1as was highly expressed in hucMSCs. CDR1as knockdown induced cell cycle arrest, cell apoptosis, and hucMSC differentiation potential impairment. These results demonstrated that CDR1as plays an important role in the stemness regulation of hucMSCs and revealed a novel role for circRNAs in hucMSCs.

## Figures and Tables

**Table 1:** Primers used in this study.

Name	Sequences (5′-3′)	Fragment size (bp)
Oct4-Forward	TTGAGGCTCTGCAGCTTAG	285
Oct4-Reverse	GCCGGTTACAGAACCACAC	
Sox2-Forward	ACACCAATCCCATCCACACT	224
Sox2-Reverse	GCAAACTTCCTGCAAAGCTC	
Sall4-Forward	TCGATGGCCAACTTCCTTC	142
Sall4-Reverse	GAGCGGACTCACACTGGAGA	
Lin28-Forward	TCGGCTTCCTGTCTATGACC	155
Lin28-Reverse	GGAATCCATCCGTGTCACTG	
Nanog-Forward	CCTGATTCTTCCACCAGTCC	292
Nanog-Reverse	TGCTATTCTTCGGCCAGTTG	
Adiponectin-Forward	GGCTATGCTCTTCACCTATG	130
Adiponectin-Reverse	TCCATTACGCTCTCCTTCC	
ALP-Forward	ATGGGATGGGTGTCTCCACA	108
ALP-Reverse	CCCACGAAGGGGAACTTGTC	
OCN-Forward	CACTCCTCGCCCTATTGGC	112
OCN-Reverse	CCCTCCTGCTTGGACACAAAG	
*β*-actin-Forward	GACCTGTACGCCAACACAGT	129
*β*-actin-Reverse	CTCAGGAGGAGCAATGATCT	
CDR1as-Forward	ACGTCTCCAGTGTGCTGA	83
CDR1as-Reverse	CTTGACACAGGTGCCATC	
circBIRC6-Forward	TCAAGGAGACCAACTTTGGC	268
circBIRC6-Reverse	CTGGAGTTTGCAGAGCAGTG	
circSMARCA5-Forward	TGGGCGAAAGTTCACTTAGAA	236
circSMARCA5-Reverse	TCTTTGCACCTCTTTCCAAAA	
circASXL1-Forward	CTCGCATGCCTCAATGCTAT	159
circASXL1-Reverse	TGCCTCTATGACCTGCAGAA	
circHIPK3-Forward	TCGGCCAGTCATGTATCAAA	155
circHIPK3-Reverse	TGCTTGGCTCTACTTTGAGTTTC	

## Data Availability

The data used to support the findings of this study are included within the article.

## Supplementary Materials

Supplementary Materials Supplementary Data1: five circRNA candidates with the highest sequencing scores in MSC from circBase Maass 2017. Figure 1: the relative expression levels of circRNAs in different cell lines.
