# Supplementary material for: The Role of CDR1as in Proliferation and Differentiation of Human Umbilical Cord-Derived Mesenchymal Stem Cells
Source: Stem Cells Int. 2019 Jun 12;2019:2316834. doi: 10.1155/2019/2316834 (PMC6594288; doi:10.1155/2019/2316834)
Supplement: Supplementary Materials — Supplementary Data1: five circRNA candidates with the highest sequencing scores in MSC from circBase Maass 2017. Figure 1: the relative expression levels of circRNAs in different cell lines. [file 2316834.f1.zip › Supplementary Figure.pdf]

Supplementary

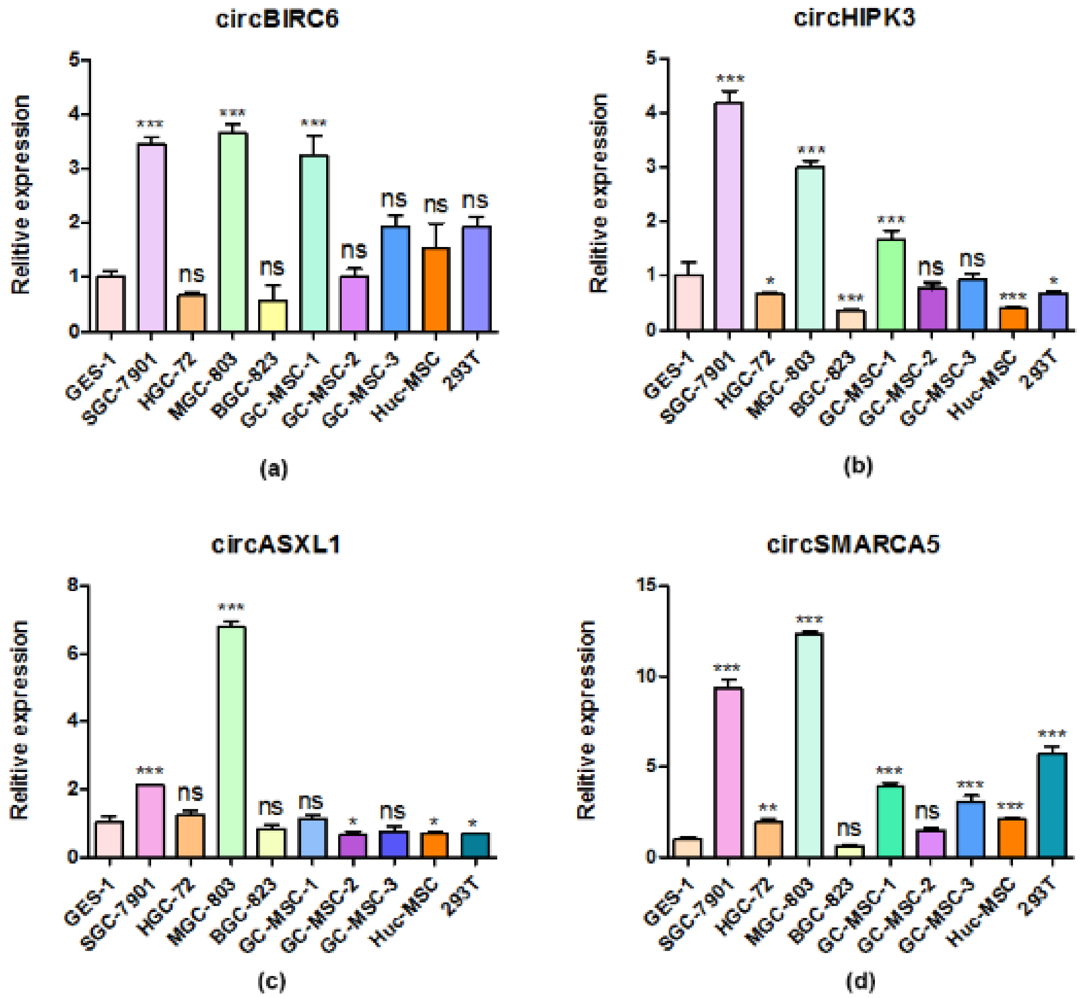

Figure 1. The relative expression levels of circRNAs in different cell lines. (a) qRT-PCR was used to detect the expression level of circBIRC6 in different cell lines; (b) qRT-PCR for detecting the circHIPK3 expression in different cell lines; (c) qRT-PCR was used to analyze the expression level of circASXL1 in different cell lines; (d) qRT-PCR for determining the circSMARCA5 expression in different cell lines. (ns, no significance; $^*P < 0.05$; $^{**}P < 0.01$; $^{***}P < 0.001$).
